# Incidental Detection of Frontal Osteoma During Pre‐Rhinoplasty Imaging

**DOI:** 10.1002/ccr3.9717

**Published:** 2024-12-07

**Authors:** Mehdi Heidarizadeh, Maryam Mohebiniya

**Affiliations:** ^1^ Department of Oral and Maxillofacial Surgery, School of Dentistry Arak University of Medical Sciences Arak Iran; ^2^ Department of Oral and Maxillofacial Radiology, School of Dentistry Arak University of Medical Sciences Arak Iran

**Keywords:** cone beam computed tomography, frontal sinus, osteoma, paranasal sinus

## Abstract

Osteoma is the most common mesenchymal tumor. Recognizing their characteristic radiographic features is crucial for early diagnosis, prompt treatment, and patient follow‐up, preventing complications. This case report describes a patient with an incidentally detected osteoma of the frontal sinus found during unrelated imaging examinations.

## 1 Case Presentation

**FIGURE 1:**
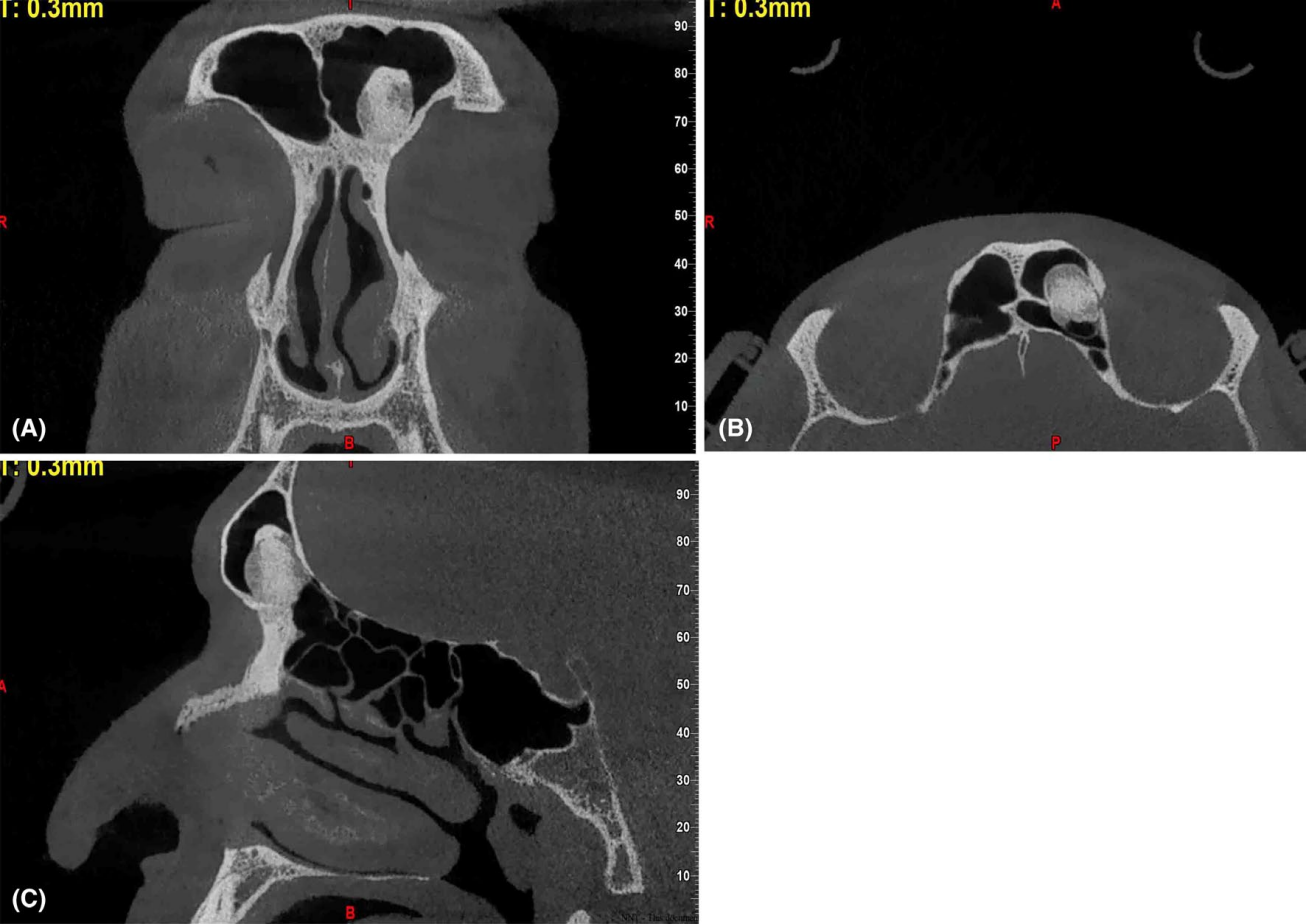
(A) Coronal and (B) axial and (C) sagittal cone‐beam computed tomography scan revealing a dense radiopaque mass in left frontal sinus.

The case we are presenting involves a 35‐year‐old male patient who was referred to a private maxillofacial radiology center for imaging before rhinoplasty surgery. The surgeon has prescribed a cone‐beam computed tomography (CBCT) radiography of the nasal septum and paranasal sinuses for preoperative evaluation. The patient reported no complications like airway obstruction or headache. He had no history of sinusitis, polyps, or previous surgery. The nose surgery was solely for cosmetic purposes. No medical history was reported, and the physical examination revealed no facial asymmetry. The evaluation of CBCT scan, including axial, coronal, sagittal, and three‐dimensional reconstructed aspects, revealed a dense bony radiopaque mass with a well‐defined border within the left frontal sinus cavity (Figure 1). The mass is attached to the anterior, posterior, and inferior walls of the frontal sinus. The size of the lesion is in the vertical dimension 16.2 mm and in the anterior–posterior dimension 12.6 mm and in the mediolateral dimension 11.7 mm.

The internal view of the lesion is radiopaque, and in some areas, a ground glass bone pattern is observed. Although the lesion has caused thinning of the supra‐medial wall of the left orbit, it has not caused involvement or any other effect on the left orbit, and the lesion has not caused thinning or expansion of the anterior–posterior wall of the frontal sinus.

Examining the radiographic features of the lesion was characteristically consistent with frontal osteoma, and definitive diagnosis of osteoma was made.

Osteoma is the most common mesenchymal tumor in the paranasal sinuses. Its common location is the frontal sinus and ethmoid air cells with a prevalence of 3.69% [1, 2]. Different etiologies such as developmental, trauma, and infection have been proposed for it, although there is still controversy regarding the exact cause. They are best diagnosed by CT, which shows a rounded, well‐circumscribed homogeneous radiodensity [2, 3].

Osteoma benign tumors are slow‐growing and often asymptomatic, typically detected incidentally in imaging studies performed for other reasons. However, symptoms like swelling, headache, and sinusitis can arise depending on the tumor's size and location (e.g., paranasal sinuses). These tumors are more prevalent in males and usually present in middle age [1, 3].

Osteoblastoma is a differential diagnosis. However, it typically affects the vertebrae and rarely involves the intracranial region [3]. As long as the osteoma does not interfere with normal function and does not cause a cosmetic problem, it does not require treatment [1]. In the case we presented, surgery was not performed due to the absence of symptoms. However, the patient was informed about the existence of the lesion, and periodic follow‐up was suggested to examine the lesion in the future. Knowledge of radiographic characteristics, understanding tumor behavior and its common location is helpful in diagnosing paranasal sinus tumors.

The distinctive radiographic characteristics of osteomas are highly diagnostic, rendering biopsy unnecessary for definitive diagnosis. Observation with serial imaging is often the mainstay of management for asymptomatic individuals. Surgical resection may be necessary for patients with troublesome symptoms.

## Author Contributions

**Mehdi Heidarizadeh:** conceptualization, project administration, writing – review and editing. **Maryam Mohebiniya:** investigation, supervision, writing – original draft, writing – review and editing.

## Consent

The patient has given his consent for his clinical information to be reported in the journal. Written informed consent was obtained from the patient to publish this report in accordance with the journal's patient consent policy.

## Data Availability

The authors have nothing to report.
